# Cannabinoid Regulation of Brain Reward Processing with an Emphasis on the Role of CB_1_ Receptors: A Step Back into the Future

**DOI:** 10.3389/fpsyt.2014.00092

**Published:** 2014-07-31

**Authors:** George Panagis, Brian Mackey, Styliani Vlachou

**Affiliations:** ^1^Laboratory of Behavioral Neuroscience, Department of Psychology, School of Social Sciences, University of Crete, Rethymno, Greece; ^2^Laboratory of Behavioural Neuroscience, School of Nursing and Human Sciences, Faculty of Science and Health, Dublin City University, Dublin, Ireland

**Keywords:** cannabinoids, endocannabinoid system, brain-reward system, intracranial self-stimulation, self-administration, conditioned place preference, reinstatement of drug-seeking behavior, CB_1_ receptors

## Abstract

Over the last decades, the endocannabinoid system has been implicated in a large variety of functions, including a crucial modulation of brain-reward circuits and the regulation of motivational processes. Importantly, behavioral studies have shown that cannabinoid compounds activate brain reward mechanisms and circuits in a similar manner to other drugs of abuse, such as nicotine, alcohol, cocaine, and heroin, although the conditions under which cannabinoids exert their rewarding effects may be more limited. Furthermore, there is evidence on the involvement of the endocannabinoid system in the regulation of cue- and drug-induced relapsing phenomena in animal models. The aim of this review is to briefly present the available data obtained using diverse behavioral experimental approaches in experimental animals, namely, the intracranial self-stimulation paradigm, the self-administration procedure, the conditioned place preference procedure, and the reinstatement of drug-seeking behavior procedure, to provide a comprehensive picture of the current status of what is known about the endocannabinoid system mechanisms that underlie modification of brain-reward processes. Emphasis is placed on the effects of cannabinoid 1 (CB_1_) receptor agonists, antagonists, and endocannabinoid modulators. Further, the role of CB_1_ receptors in reward processes is investigated through presentation of respective genetic ablation studies in mice. The vast majority of studies in the existing literature suggest that the endocannabinoid system plays a major role in modulating motivation and reward processes. However, much remains to be done before we fully understand these interactions. Further research in the future will shed more light on these processes and, thus, could lead to the development of potential pharmacotherapies designed to treat reward-dysfunction-related disorders.

## Introduction

Cannabis is considered as one of the oldest and most widely used recreational drugs in the world. Its consumption has increased dramatically in recent decades along with questions of its categorization as an illegal substance (1–4). The attraction of cannabis and the many issues surrounding its illegality stem from its effects on sensory processing, euphoric sensations, and its relaxing inferences. These effects are mainly attributed to the key psychoactive ingredient of marijuana, Δ^9^-tetrahydrocannabinol (Δ^9^-THC) (5–8). The effects of this psychoactive component lead to drug-seeking behavior and drug abuse in humans (1, 4). Conversely, investigation of the rewarding effects of Δ^9^-THC and other synthetic cannabinoids in animal models of drug abuse and dependence has provided us with valuable information on the biphasic effects of these compounds through contradictory findings (9–11). The discovery of the endogenous cannabinoid system has fueled the progressing amount of cannabinoid research in recent years, with particular emphasis on the effects of endogenous and synthetic cannabinoid compounds on cannabinoid 1 receptors (CB_1_ receptors) found in different areas of the brain. This system is thought to modulate the motivational processes and reward-seeking behaviors associated with the use of cannabis. Hence, the present review summarizes recent animal studies that investigate the function of the endocannabinoid system and its involvement in brain-reward systems, with particular emphasis on the role of CB_1_ receptors.

## Endogenous Cannabinoid System

### Definition

The endogenous cannabinoid or endocannabinoid system was first identified in the early 1990s when researchers were trying to shed light on the mechanisms of action of Δ^9^-THC (12–15). For the past couple of decades, with the contribution of various research groups, it has been discovered that the endocannabinoid system is composed of cannabinoid 1 and 2 receptors (CB_1_, CB_2_, respectively, and possibly others), endogenous ligands for these receptors and enzymes responsible for the synthesis, reuptake and degradation of these endogenous ligands (14, 16, 17). Genetic, pharmacological, and behavioral methods have all been utilized in order to elucidate the function and mechanisms of this system.

### Cannabinoid receptors

The discovery of Δ^9^-THC has resulted in a wealth of research surrounding cannabinoid receptors. Further, the discovery of synthetic cannabinoid agonists with the ability to simulate the effects of Δ^9^-THC suggested the existence of specific cannabinoid receptors (18) and increased our understanding of the mechanisms of action of Δ^9^-THC and the function of cannabinoid receptors. Two cannabinoid receptors have so far been identified, CB_1_ (19, 20) and CB_2_ receptors (21), both of which are metabotropic receptors coupled to Gi/o proteins. CB_1_ receptors are observed throughout the central and peripheral nervous system, but with higher concentrations in the brain and spinal cord (22). This convergence of CB_1_ receptors in the central nervous system (CNS) is consistent with the studied behavioral and physiological effects of cannabinoids (23). High levels of these receptors are found in brain areas such as the hippocampus, which may explain the memory deficits associated with the use of cannabis. Similarly, a high concentration of these receptors is also observed in brain areas, such as the basal ganglia and cerebellum, associated mainly with motor function and coordination (24, 25). The mesocorticolimbic dopaminergic pathway of the brain similarly features a high amount of CB_1_ receptors. Brain areas that are part of the mesocorticolimbic dopaminergic pathway include the prefrontal cortex, the hippocampus, the olfactory bulb, and the nucleus accumbens, all of which are implicated in motivational and reward processes, which have also been found to be altered by cannabinoid compounds (26, 27). CB_1_ receptors are also thought to inhibit release of glutamate, GABA, and other neurotransmitters, such as dopamine (28). More recent evidence suggests that CB_2_ receptors are also implicated in the moderation of cannabinoids in the CNS (28). Further, a number of behavioral and pharmacological effects of cannabinoid compounds cannot be explained by their action specifically on CB_1_ and CB_2_ receptors, proposing the existence of additional cannabinoid receptors, further to be identified and characterized (29, 30).

### Endocannabinoid ligands and their metabolizing enzymes

The discovery of cannabinoid receptors alludes to the existence of endogenous ligands that bind and impact the function of these receptors. The two most widely studied endocannabinoids are *N*-arachidonoylethanolamide (AEA), also called anandamide (31) and 2-arachidonoylglycerol (2-AG) (32, 33), which were first discovered in the early 1990s. Endocannabinoids are synthesized on demand, mainly postsynaptically and act as retrograde messengers regulating the presynaptic release of neurotransmitters (34). This occurs in response to physiological and pathological stimulus resulting after an increase of the intracellular concentration of Ca^2+^ (35). Different pathways are involved in the synthesis of AEA and 2-AG. AEA is formed by transacylation of phosphatidylinositol and subsequent degradation by the phospholipid precursor *N*-acetyl-phosphatidylethanolamine (NAPE), as well as via a pathway involving the phospholipase C (PLC)-catalyzed cleavage of NAPE to generate a lipid, phosphoanandamide, which is subsequently dephosphorylated by phosphatases (36, 37). Although several pathways have been proposed for 2-AG synthesis, the one which dominates in the CNS involves the production of 2-AG via a two-step process: degradation of arachidonate-containing phospholipids to diacylglycerol (DAG) by PLC followed by DAG lipase-catalyzed degradation to 2-AG (38). AEA and 2-AG activate both CB_1_ and CB_2_ receptors. These endogenous ligands emulate many behavioral and biochemical properties of cannabinoids (36, 39). In the case of AEA, activation of the transient receptor potential vanilloid type 1 (TRPV1) receptor has also been noted (40). In recent years, more endocannabinoid ligands have been discovered such as *N*-arachidonoyldopamine, virodhamine, and noladine ether (41, 42). However, the physiological effects of these endocannabinoids are yet to be revealed. Thus, the focus of our review will be on effects of AEA and 2-AG, as these are the first two endocannabinoids discovered and mostly studied. Endocannabinoids are present in the mesolimbocortical dopaminergic system of the brain (24) suggesting an association with motivation and reward (31). The control of rewarding processes seems to be mainly moderated by CB_1_ receptors. Endocannabinoids can passively diffuse through lipid membranes, but a highly affinity transporter, which is not yet identified, seems to accelerate this process. Finally, two types of metabolizing enzymes seem to play a role in endocannabinoid deactivation, a fatty acid amide hydrolase (FAAH) is the main hydrolase for AEA, whereas 2-AG inactivation is mainly degraded by two other enzymes, called monoacyl-glycerol lipases (MAGLs) (34).

## Pharmacological Modulation of the Endocannabinoid System

The discovery of the endocannabinoid system has led to the synthesis of agonists and antagonists that have proven useful in the investigation of CB_1_ and CB_2_ receptors and their functions. Such pharmacological modulation of the endocannabinoid system has led to recent advances in behavioral and pharmacological research (43, 44). There are currently five classes of cannabinoid analogs that have been classified based on their structure (45–47). These classes are classical, non-classical, aminoalkylindoles, eicosanoids, and biarylpyrazoles.

Classical cannabinoids are tricyclic terpenoid derivatives. This group includes the main psychoactive component of cannabis Δ^9^-THC, the phytocannabinoid Δ^8^-THC, and other synthetic equivalents. Levonantradol and AMG-3 are two examples of cannabinoid compounds belonging to this class (43, 44, 48–50).

Non-classical cannabinoids incorporate bicyclic and tricyclic analogs of Δ^9^-THC. These include, among others, the potent non-selective CB_1_/CB_2_ receptor agonists CP-55,940, CP-47,497, and CP-55,244 (43, 51).

The eicosanoid group consists of CB_1_ and CB_2_ receptor agonists that have markedly different structures not only from aminoalkylindoles but also from classical and non-classical cannabinoids. Notable members of this group are the endocannabinoids AEA and 2-AG (33, 52).

Aminoalkylindoles have a completely different chemical structure from other classes of cannabinoids. They are less lipophilic and differ in the way they interact with cannabinoid receptors (32). The highly studied WIN 55,212-2 is a member of this class of cannabinoids. It has high stereoselectivity, but low affinity for the CB_2_ receptor (48).

There are many compounds that selectively activate CB_1_ receptors more effectively than CB_2_ receptors. Many of these are synthetic analogs of AEA, which include R-(+)-methanandamide, arachidonyl-2′-chloroethylamide (ACEA), and arachidonylcyclopropylamide (ACPA) (48, 53). Although this is not the focus of this review, compounds with a selective affinity for CB_2_ receptors have also been developed and feature cannabinoids such as JWH-133, L-759633, and L-759656, and the non-classical cannabinoid HU-308. Other selective CB_2_ receptor agonists are the aminoalkylindoles JWH-015 and AM1241 (43, 44, 48).

Many selective compounds have been used extensively in research as CB_1_ receptor competitive antagonists. Two of the most well-known members of this group are SR141716A (rimonabant), AM-251, as well as AM-281 and LY320135 (47, 54, 55). These cannabinoids have greater affinity for CB_1_ receptors than for CB_2_ receptors and can also inhibit agonist-induced activation of CB_1_ receptors. In some cases, however, these can act as inverse agonists (47). Recently, compounds have been developed that act as CB_1_ receptor antagonists, yet they do not induce signs of inverse agonism at these receptors. Cannabinoids such as NESS O327, O-2050, and AM4113 show such effects (47, 56). In addition, the compounds AM630 and SR144528 are stronger in blocking CB_2_ than CB_1_ receptor activation (57, 58). However, both are considered to be CB_2_ receptor inverse agonists, due to the fact that, when administered alone, they can cause inverse cannabimimetic effects in CB_2_ receptor-expressing tissues (59).

Other cannabinoids that show an affinity for CB_1_ and/or CB_2_ receptors are the phytocannabinoids cannabinol, cannabidiol, and cannabigerol. Cannabinol acts as a CB_1_ receptor partial agonist, yet there is evidence to suggest that it can also serve as a CB_2_ receptor agonist/inverse agonist (60). Cannabidiol and cannabigerol have been shown to act as CB_1_ receptor antagonists/inverse agonists. Furthermore, cannabidiol has been found to have considerable potency as a CB_2_ receptor antagonist/inverse agonist (61). Recent research has indicated that the actions of AEA and 2-AG are halted by cellular uptake and intracellular enzymatic hydrolysis. This has been highlighted by the synthesis of several drugs that inhibit these actions (62–64). The use of these drugs as tools in animal experiments has elucidated the pathophysiological actions of endocannabinoids. Significant members of this group include the FAAH inhibitors/indirect agonists PMSF, palmitylsulphonyl fluoride (AM374), stearylsulphonyl fluoride (AM381), O-1887, OL-135, URB-532, URB-597, and URB-602 (65–67). In the last few years, selective pharmacological tools that disrupt the activity of MAGL *in vivo* have also become available. MAGL activity is sensitive to general serine hydrolase inhibitors, such as PMSF. However, as such compounds also inhibit FAAH, they are not suitable to distinguish the function of these enzymes. More selective compounds include URB602, NAM, OMDM169, JZL184, and KML29 (68).

There is some pharmacological evidence that points toward the existence of the reuptake transporter of endocannabinoids through the use of specific reuptake inhibitors. Amongst these reuptake inhibitors, AM-404 is the most widely investigated. However, this compound not selective, as it also halts the action of FAAH and binds to CB_1_ receptors (67).

## Genetic Modulation of the Endocannabinoid System

Transgenic mice have been used in recent research to understand the pharmacological and behavioral actions of cannabinoids [for details on genetic modulation of the endocannabinoid system, please see Ref. (69–71)]. These mice lack CB_1_, CB_2_, or both CB_1_ and CB_2_ receptors. They have proven useful tools to elucidate whether responses to cannabinoid compounds are attributed to CB_1_ receptors and/or CB_2_ receptors as well as the physiological roles of these receptors (70, 71). FAAH- and MAGL-deficient mice are also useful in understanding the physiological role of these endocannabinoid components in various functions and disorders, including brain reward and drug addiction (68, 72). However, several adaptive changes in CB_1_ receptor function have been reported in MAGL knockout mice, limiting the use of these mutants in behavioral studies. Recently, a novel line of transgenic mice that overexpress MAGL in the forebrain has been generated. Since these mice do not express adaptive changes in other endocannabinoid components, this opens the possibility to expand the study of the physiological role of 2-AG in brain reward processes and drug addiction (73).

## Cannabinoid Effects on Brain Reward Processes

### Cannabinoid effects on brain-stimulation reward

Intracranial self-stimulation (ICSS) is an operant behavioral paradigm in which animals would work to obtain intracranial stimulation through electrodes implanted into discrete brain areas (often referred as brain reward areas/circuit) (74, 75). This observation is based on the original discovery by Olds and Milner (76) that rats will repeatedly press a lever to stimulate components of their brain reward circuit. Historically, ICSS has been utilized in rodents to study how pharmacological or molecular manipulations affect brain reward function (77). More importantly, manipulations that increase reward and manipulations that decrease reward produce opposite outputs in self-stimulation behavior. Accordingly, most drugs of abuse are able to lower ICSS threshold (i.e., increase the rewarding efficacy of intracranial stimulation), which support the notion that they activate the same substrate with electrical stimulation in a synergistic manner (78–80). Thus, ICSS can be considered as a model to study the reward-facilitating effects of various drugs of abuse with addictive properties in humans.

Over the last years, a considerable amount of literature has been published on the effects of cannabinoids in the ICSS paradigm (see Table 1). Importantly, different effects have been observed after the administration of Δ^9^-THC or other CB_1_ receptor agonists and endocannabinoid modulators. Overall, the corresponding findings appear to be dispersed and dependent on various methodological variables (i.e., strain of the animal, cannabinoid compound, and dose).

**Table 1:** **Cannabinoid effect on intracranial self-stimulation in experimental animals**.

Cannabinoid drug	Dose	Effect	Species	Reference
Δ^9^-THC, nabilone, canbisol	0.12–10 mg/kg	↑Threshold	Long-Evans rats	(81)

Levonantradol	0.2, 0.3 mg/kg	↑Threshold	Albino CDF rats	(82)

Δ^9^-THC	1.5 mg/kg	↓Threshold	Lewis rats	(83)

Δ^9^-THC	1 and 1.5 mg/kg	↓Threshold	Lewis rats	(84)

Δ^9^-THC	1 mg/kg	–	Sprague-Dawley rats	(85)
		–	Fischer 344 rats	
		↓Threshold	Lewis rats	

CP 55,940	10, 25, 50 μg/kg	–	Lewis rats	(86)

SR141716A	1, 3, 10 mg/kg	↑Threshold	Sprague-Dawley rats	(87)

WIN 55,212-2	0.1, 0.3 and 1 mg/kg	↑Threshold	Sprague-Dawley rats	(88)

WIN 55,212-2	0.1, 0.3, 1, 3 mg/kg	↑Threshold	Sprague-Dawley rats	(89)
CP 55,940	10, 30, 56, 100 μg/kg			
HU-210	10, 30, 100 μg/kg			
SR141716A	0.02 mg/kg	(Reversing effect on agonists)		

AMG-3	1, 2, 4, 8 mg/kg	↑Threshold	Sprague-Dawley rats	(90)

PMSF	15, 30, 60 mg/kg	↑Threshold	Sprague-Dawley rats	(91)
OMDM-2	3, 10, 30 mg/kg			
URB-597	0.3, 1, 3 mg/kg			
SR141716A	0.02 mg/kg	(Reversing effect on modulators)		

SR141716A	0.1–10 mg/kg	↑Threshold	CB1-knock out mice	(92)

AM251	0.1–10 mg/kg	–	CB1-knock out mice	(92)

Δ^9^-THC	1–2 mg/kg	↑Threshold (reversing effect on Δ^9^-THC)	Sprague-Dawley rats	(93)

SR141716A	0.02 mg/kg			(93)

AM-251	3 mg/kg	↓Opportunity cost	Long-Evans rats	(94)

Δ^9^-THC	0.32–1 mg/kg	–	Sprague-Dawley rats	(95)
SR	3.2 and 10 mg/kg	↑Threshold		
CP	1 mg/kg	– (But reversed THC effects)		
	0.01–0.032 mg/kg	–		
	0.1 and 0.32 mg/kg	↑Threshold		

Δ^9^-THC	0.1 mg/kg	↓Threshold	Sprague-Dawley rats	(96)
	1 mg/kg	↑Threshold		

URB-597	1 mg/kg	–	Sprague-Dawley rats	(97)
	3.2 and 10 mg	↑Threshold		

*– No effect on threshold, ↑ increase, ↓ decrease, – no effect*.

A number of studies have been conducted on the effects of Δ^9^-THC in the ICSS paradigm. Gardner and colleagues were among the first who studied the effects of Δ^9^-THC on ICSS. In their experiments, 1 and 1.5 mg/kg of Δ^9^-THC decreased ICSS thresholds in Lewis rats, but not in Fisher 344 rats, whereas in Sprague-Dawley rats the effect was only marginal (83, 85). In contrast, other studies failed to show an enhancement of brain-stimulation reward with Δ^9^-THC in the dose range from 0.5 to 10 mg/kg in Sprague-Dawley rats under baseline conditions (93, 95, 96) or in animals pre-exposed to stress (98). Similar results have been reported in Long-Evans rats with p.o administration of 10/mg/kg Δ^9^-THC and various doses of three synthetic analogs structurally related to Δ^9^-THC, namely levonantradol, nabilone, and canbisol (81, 82). Interestingly, however, in a recent study from our research group, we showed that Δ^9^-THC can induce both rewarding and anhedonic effects in the ICSS paradigm in Sprague-Dawley rats, depending on the dose used (96). Thus, a low dose of 0.1 mg/kg, decreased ICSS thresholds and caused clear parallel leftward shifts in the rate-frequency function, whereas a higher dose of 1 mg/kg increased ICSS thresholds, producing rightward shifts. These effects were long-lasting, since they remained for 2 h post-injection and the reward-facilitating effect that we observed with 0.1 mg/kg of Δ^9^-THC was more pronounced after 1 h. Both the rewarding and the anhedonic effects of Δ^9^-THC observed in our studies are specifically mediated by cannabinoid CB_1_ receptors, since they have been reversed by a low dose of SR141716A. Comparing findings from the above studies, it can, thus, be suggested that Lewis rats may have a differential sensitivity to Δ^9^-THC, compared to Sprague-Dawley and Fisher 344 rats and that the dose–response function of Δ^9^-THC on brain-stimulation reward is not linear, but rather biphasic.

Only a few studies have examined the effects of various synthetic cannabinoid agonists on brain-stimulation reward. Arnold and colleagues have reported that the potent synthetic CB_1_ receptor agonist CP55,940 did not affect the reinforcing efficacy of medial forebrain bundle (MFB) stimulation (86). In the same way, other studies have shown that the synthetic CB_1_ receptor agonists WIN55,212-2, CP55,940, HU-210, and AMG-3 either do not affect or increase ICSS threshold, depending on the dose used (88–90, 95). Similarly, in a series of studies from our laboratory we have shown that the indirect cannabinoid agonists (endocannabinoid modulators) PMSF, AM-404, OMDM-2, and URB-597 in low doses do not affect ICSS thresholds, while in high, and possibly non-selective doses, decrease the reinforcing efficacy of brain stimulation (91, 99). Similar results have been reported very recently with the FAAH inhibitor URB-597 (97).

Several studies have examined the effects of CB_1_ receptor antagonists on ICSS. Low doses of the CB_1_ receptor antagonists SR141716A and AM-251 did not affect ICSS thresholds (89–92, 95), while higher doses of SR141716A have been reported to increase ICSS thresholds (86, 87, 92) However, in such high doses it is possible that SR141716A acts as a partial or inverse agonist at cannabinoid receptors, as it has been observed in other studies (100, 101). Indeed, this could be a plausible explanation for its anhedonic effects observed with high doses on brain-stimulation reward. Shizgal’s group (94) utilizing a novel method for measuring reward have shown that AM-251 decreased performance for MFB self-stimulation. Indeed, AM-251 produced leftward shifts of the function that relates operant performance to the opportunity cost of the reward, but did not affect the function that relates operant performance to the stimulation strength. The authors suggest that this shift may be related to a decrease in the reward signal gain or an increase in the subjective reward cost.

In summary, although most drugs abused by humans are able to increase the rewarding efficacy of brain stimulation over a wide range of doses, results with Δ^9^-THC and other synthetic cannabinoid agonists have not always been consistent. In the studies by Gardner’s group, the most robust reward-facilitating effect of Δ^9^-THC in the ICSS paradigm was found in rats of the Lewis strain. Thus, it is possible to hypothesize that the reward-facilitating effect of Δ^9^-THC may preferentially be obtained in certain strains of rat, suggesting an important genetic component in this action. One major finding was that Δ^9^-THC induces biphasic effects, i.e., is able to induce both rewarding and anhedonic effects, in the ICSS paradigm in Sprague-Dawley rats, depending on the dose used. On the other hand, studies using the ICSS paradigm failed to show any reward-facilitating effects for direct and indirect (i.e., endocannabinoid modulators) synthetic cannabinoid agonists, or to the contrary, they present data for anhedonic actions of these compounds. Thus, it is possible that cannabinoids have negative or dysphoric effects in animals that mask their reward-facilitating effects in the ICSS paradigm and that these effects are suppressed under a limited dose range.

### Cannabinoids effects on conditioned place preference

Conditioned place preference (CPP) is a non-operant procedure for assessing the reinforcing properties of drugs using a Pavlovian conditioning. The reinforcing properties of abused drugs are easily associated with environmental stimuli, such as an environment or context in which the drugs are administered. Through multiple pairings, these environmental (contextual) cues acquire conditioned reinforcing properties. The CPP paradigm is based on the assumption that animals learn to approach stimuli paired with rewards and to avoid stimuli paired with aversive agents. Thus, it can be used to evaluate whether the repeated pairing of one specific environment with a drug produces a preference for that environment (102). Indeed, in this procedure, the animal develops an association between the subjective state produced by the drug (e.g., a heightened feeling of euphoria comparable to pleasure in humans) and the environmental cues present during the drug state. Most drugs abused by humans produce place preference in experimental animals (103). Although CPP provides a less direct evaluation of the rewarding effects of drugs, it presents several advantages: (1) it can be sensitive even to low doses of the drug studied, (2) it can be also used to assess the aversive or dysphoric properties of a drug (in this case, the animal will avoid staying in a compartment previously associated with a drug), (3) the animals are tested in a drug-free state, (4) it can be used to study non-drug stimuli, such as food, sucrose, or sex.

Studies using the CPP paradigm have shown that Δ^9^-THC and other synthetic cannabinoid agonists can induce both appetitive and aversive effects under various experimental conditions (see Table 2). Notably, in the studies reporting place preference of cannabinoids, these effects are usually dependent upon the particular dose used and the preference is connected to a single dose. Furthermore, other factors, such as the administration of a priming injection and the timing between injections have been suggested to be important in determining whether cannabinoids produce preference or aversion.

**Table 2:** **Cannabinoid effects on conditioned place preference in experimental animals**.

Cannabinoid drug	Dose	Effect	Species	Reference
Δ^9^-THC	1 mg/kg	–	Long-Evans rats	(104)
	2 and 4 mg/kg	CPP		
	2 and 4 mg/kg (wash-out period)	CPA		
	1 mg/kg	CPP		

Δ^9^-THC	1 mg/ml	CPA	Lewis and Sprague-Dawley rats	(105)

CP 55,940	100 μg/kg	CPA	Wistar rats	(106)

Δ^9^-THC	1.5 mg/kg	–	Sprague-Dawley rats	(107)
	15 mg/kg	CPA		

WIN 55,212-2	0.3–1 mg/kg	CPA	Wistar rats	(108)
SR141716A	Up to 10 mg/kg	–		
		(reversing effect on WIN 55,212-2)		

Δ^9^-THC	20 mg/kg	CPA	CD1 mice	(109)

Δ^9^-THC	1 and 1.5 mg/kg	CPA	Wistar rats	(110)
Anandamide (AEA)	up to 16 mg/kg	-		

HU-210	20, 60, 100 μg/kg	CPA	Lister Hooded rats	(111)
Δ^9^-THC	1.5 mg/kg	CPA		

Δ^9^-THC	5 mg/kg	CPA	CD1 mice	(112)
	1 mg/kg	–		
	5 mg/kg (not standard protocol-pre-treatment)	–		
	1 mg/kg	CPP		

CP 55,940	20 μg/kg	CPP	Wistar rats	(113, 114)
SR141716A	0.5 mg/kg	–		
		(Reversing effect on CP 55,940)		

Δ^9^-THC	5 mg/kg	-	dynorphin deficient mice	(115)

Δ^9^-THC	2 mg/kg	CPA	Lister Hooded rats	(116)
WIN 55,212-2	1 and 3 mg/kg	–		

Δ^9^-THC	0.075–0.75 mg/kg	CPP	Wistar rats	(117)
SR141716A	0.25–1 mg/kg	(Reversing effect on Δ^9^-THC)		

WIN 55,212-2	mg/kg (+pre-treatment)	CPP	CD1 mice	(118)
	1 mg/kg (+pre-treatment)	–		

Δ^9^-THC	1 mg/kg	CPP	A_2A_ KO and wild-type mice	(119)
	5 mg/kg	CPA		

URB-597	0.03–0.3 mg/kg	–	Wistar, Sprague-Dawley rats	(120)

AM-404	1.25–10 mg/kg	CPP	Rats (anxiety models)	(121)
		–		

Δ^9^-THC	0.1 mg/kg	CPP	Sprague-Dawley rats	(122)

Δ^9^-THC	1 mg/kg	–	C57B1/6Lx129Sv mice	(93)

Anandamide	0.03–3 mg/kg, iv	–	Sprague-Dawley rats	(123)
WIN 55,212-2	0.3 and 3 mg/kg, iv (+URB-597 0.3 mg/kg, ip)	CPA		
AM-251	50, 150, and 300 mg/kg, iv	CPA		
	3 mg/kg, ip	–		
		(Reversing effect on anandamide and WIN 55,212-2		

Δ^9^-THC	1 mg/kg	–	ICR mice	(124)
	10 mg/kg	CPA		

Δ^9^-THC	5 mg/kg	–	Adolescent Wistar rats	(125)
	5 mg/kg	CPA	Adult Wistar rats	

WIN	0.25, 1.25, 2.5 mg/kg	CPA	Adult Wistar rat	(126)
	0.25 mg/kg	CPP	Adult SHR rats	
	2.5 mg/kg	CPP	Adolescent SHR rats	

AM-251	0.25 and 0.5 mg/kg	–	Wistar rats	(127)

SR 141716A	0.5, 1 and 2 mg/kg	–	Wistar rats	(128)

WIN 55,212-2	0.5 mg/kg	CPP	OF1 male mice	(129)
	0.1 mg/kg	–		

Intra-accumbal SR141716	0.5 μg/μl	–	Wistar rats	(130)
	1.5 μg/μl	CPP		

HU-210	100 μg	CPP	Sprague Dawley rats	(131)

Δ^9^-THC	10 mg/kg	–	Albino Wistar rats	(132)

SR 141716A	0.3 and 3 mg/kg	–	Sprague Dawley rats	(133)

HU-210	100 μg	CPP	Sprague Dawley rats	(134)

Intra-CeA ACPA	5 ng	CPP	Wistar rats	(135)
Intra-CeA AM-251	120 ng	CPA		

AM-404	1.25 mg/kg	–	Sprague Dawley rats	(136)
	10 mg/kg	CPP		
Δ^9^-THC	0.1–3 mg/kg	–		

Intra-VTA ACPA	0.5 and 1 ng	CPA	Wistar rats	(137)
Intra-Bla ACPA	1 and 2 ng	CPP		
Intra-VH ACPA	3 ng	CPA		
	6 ng	CPP		

Intra-accumbal WIN 55,212-2	1, 2, 4 mmol/0.5 μl	CPP	Wistar rats	(138)
Intra-accumbal AM-251	90 μmol/0.5 μl	CPA		

Intra-VTA WIN 55,212-2	4 mmol/0.3 μl	CPP	Wistar rats	(139)
Intra-VTA AM-251	90 mmol/0.3 μl	Tendency (not significant effect) toward CPA		

JWH-018	0.1 and 1 mg/kg (in drug naïve mice)	CPA	NIH Swiss mice	(140)
	0.1 mg/kg (in mice pretreated with Δ^9^-THC)	CPP		

WIN 55,212-2	1 mg/kg	–	Sprague Dawley rats	(141)
Δ^9^-THC	0.5 mg/kg	–		
		(Tendency for CPA)		

*CPP conditioned place preference, CPA conditioned place aversion (avoidance), – no effect*.

In the first study, Δ^9^-THC-produced CPP was published in 1995 by the Gardner research group (104). In their experiments, 2–4 mg/kg Δ^9^-THC produced CPP in Long-Evans rats, when the CPP pairing interval was 24 h, while the dose of 1 mg/kg of Δ^9^-THC did not produce any preference. However, when the schedule of daily injections was changed, allowing a longer wash-out interval between injections (48 h), Δ^9^-THC produced a clear place aversion in the dose range of 2–4 mg/kg, but place preference in the dose of 1 mg/kg. In other words, Δ^9^-THC-induced CPP was dependent upon the dose and the injection schedule. Similar results have been reported by Braida and colleagues (117) and Le Foll and colleagues (122). In the first study, Δ^9^-THC produced CPP in Wistar rats in a dose range between 0.075 and 0.75 mg/kg, whereas higher doses produced aversive effects. In the latter study, a low 0.1 mg/kg dose of Δ^9^-THC produced CPP, while doses lower or higher than this did not produce any preference. Two other studies in male Lister-Hooded rats not only failed to find any rewarding effects of Δ^9^-THC (1.5 and 2 mg/kg) in the CPP paradigm, but reported aversive effects (111, 116). Two more recent studies did not find any rewarding or aversive effects of Δ^9^-THC in the CPP paradigm in Sprague-Dawley rats and in the dose range of 0.1–3 mg/kg (136, 141). A few studies have examined whether adolescent rats respond differently (are more vulnerable) to Δ^9^-THC than adult rats. According to a study by the McGregor group (125), Δ^9^-THC (5 mg/kg) produced CPA in adult rats, whereas in adolescent rats there was only a trend toward aversion, which was not significant. Interestingly, the aversive effect reported in adult rats was long-lasting, since the animals still avoided Δ^9^-THC-paired environment 16 days following the last drug exposure. Surprisingly, in a more recent study in adolescent Wistar rats, although Δ^9^-THC (10 mg/kg) did not induce CPP when administered alone, it tended to produce a preference when administered in combination with cannabidiol (132).

Studies in mice have also shown controversial results. Valjent and Maldonado reported Δ^9^-THC-induced CPP bypassing the dysphoric/aversive effects of Δ^9^-THC that has been reported in naïve animals with a priming injection 24 h before the first conditioning session (112). However, Vlachou and colleagues did not observe CPP with the 1 mg dose of Δ^9^-THC using the same experimental manipulation (93). These differences can be explained in part by the different strain of animals used, the number of pairings or the periods of conditioning, and administration of the drugs. Although the Maldonado group replicated their findings in a subsequent study (119), it is worth noting that they also report conditioned place aversion (CPA) with the dose of 5 mg/kg of Δ^9^-THC. Finally, a number of studies have found that Δ^9^-THC produces CPA and not CPP in rats or mice (105, 107, 109, 110, 124).

Bidirectional and/or conflicting effects have been also reported in the literature for synthetic cannabinoid agonists. In a very recent study, the synthetic cannabinoid agonist JWH-018 produced CPA in naïve mice, but CPP in mice pre-treated with Δ^9^-THC (140). Thus, we could speculate that Δ^9^-THC pre-exposure may reveal the appetitive effects of other cannabinoid agonists. Braida and colleagues using the potent synthetic CB_1_ receptor agonist CP55,940 in Wistar rats reported CPP only at the dose of 20 μg/kg, but not in lower or higher doses (113). Another study using CP55,940 reported CPA in the dose of 10 μg/kg, as well as in a higher dose of 100 μg/kg (106). In the same study, the dose of 100 μg/kg of CP55,940 was also aversive in the conditioned taste aversion paradigm. The literature on the reinforcing effects of WIN55,212-2 and HU-210 in the CPP paradigm is also controversial. According to Castané and colleagues, WIN55,212-2 produced CPP in mice pre-exposed to a priming injection of the drug (118). Similar results have also been reported with HU-210 (100 μg/kg) in Sprague-Dawley rats pre-exposed to a priming phase consisting of four daily home injections of the drug (131, 134). Notably, CPP with WIN55,212-2 has also been reported in OF1 mice without utilizing a pre-exposure protocol (129). However, two other studies reported CPA after systemic administration of WIN55,212-2 (108) and HU-210 (111). Adding to this complexity are studies that failed to reveal either a preference or an aversive effect with WIN55,212-2 in a dose range between 0.1 and 3 mg/kg (116, 142). Contrasting effects of WIN55,212-2 in Wistar and spontaneously hypertensive rats, a validated animal model of attention deficit/hyperactivity disorder, have been reported in the literature (126). Thus, WIN55,212-2 produced CPA only in adult, but not adolescent Wistar rats. In contrast, WIN55,212-2 produced CPP in both adolescent and adult spontaneously hypertensive rats.

A limited number of studies have also examined the effect of intracranial injections of CB_1_ receptor agonists and antagonists in the CPP paradigm. Data from two recent studies have shown that intra-accumbal (138) and intra-VTA (139) injection of WIN55,212-2 produces CPP. In contrast, intra-accumbal (138) or intra-central amygdala (135) injection of the CB_1_ receptor antagonist AM-251 produces CPA, while intra-VTA injection of AM-251 produces a tendency toward CPA (139). Similarly, intra-central amygdala (135) and intra-basolateral amygdala injection of the cannabinoid agonist ACPA produces CPP, whereas intra-VTA injection of ACPA produces CPA (137). Interestingly, biphasic effects of intra-ventral hippocampus injection of ACPA have also been reported in the literature in the CPP test, with lower doses producing CPP, while higher doses CPA (137).

Although most of the studies have used CB_1_ receptor antagonists to test for CB_1_-receptor selectivity of cannabinoid compounds on brain reward, there are a few studies that have tested the effects of CB_1_ receptor antagonists on reward *per se*. Cheer and colleagues found that the CB_1_ receptor antagonist/inverse agonist SR141716A produced a clear CPP (111), indicating the possibility that an endogenous cannabinoid tone might be present in the brain, as a physiological system to suppress reward or induce aversion. Importantly, in a major study, intra-accumbens injection of SR141716A also produces CPP, although *in vivo* silencing of accumbal CB_1_ receptors induced CPA to cocaine (130). Based on these results, the authors suggest that SR141716A acts as an inverse agonist on the CPP test. However, in other studies SR141716A or AM-251 failed to produce either CPP or CPA (108, 113, 127, 128, 133).

A limited number of studies have examined the effects of endogenous cannabinoids or compounds increasing their levels in the brain on CPP. The first report that the administration of the endogenous cannabinoid anandamide did not produce any significant effects in place conditioning was published by Mallet and Beninger (110). As anandamide is quickly degraded, its physiological roles can be best studied by blocking the mechanisms of its degradation and, thus, prolonging its actions. As previously described, anandamide degradation is mainly mediated by the enzyme FAAH. Accordingly, inhibition of FAAH by drugs, such as URB-597, can be used as a pharmacological tool to study the role of anandamide in brain reward. In a study investigating the antidepressant properties of URB-597, Gobbi and colleagues (120) did not find any rewarding effects in the CPP paradigm. In another major study (123), intravenous administration of anandamide did not produce CPP or CPA. However, when rats were pretreated with the FAAH inhibitor, URB597 anandamide produced dose-related CPA (123).

As mentioned previously, termination of endocannabinoid signaling is also mediated by cellular uptake. Inhibition of endocannabinoid transport by drugs, such as AM-404, is an additional pharmacological tool to study the role of endocannabinoids on brain reward. CPP by AM-404 was first demonstrated experimentally by Bortolato and colleagues (121) in rats housed under enriched conditions, but not in rats kept in standard cages. However, it is worth noting that AM-404 induced CPP at a dose that did not increase tissue levels of anandamide or 2-AG in the brain areas investigated (121). Thus, the involvement of the endocannabinoid system in AM-404-induced CPP remains questionable. In a more recent study, Scherma and colleagues examined different doses (1.25–10 mg/kg) of AM-404 in the CPP test and found that only the high dose of 10 mg/kg was able to produce a clear CPP in Sprague-Dawley rats (136).

In summary, while almost all drugs abused by humans are able to produce a clear and reliable place preference (i.e., increase the time spent in the drug paired compartment) over a range of doses, results with Δ^9^-THC and other cannabinoids have not always been consistent. The studies reporting a CPP associated with administration of a cannabinoid have either used a particular experimental methodology or the preference occurred at only a single dose. In addition, although endocannabinoids are able to regulate reward-related processes, they do not produce CPP and do not seem to have reinforcing properties that have been associated with Δ^9^-THC and other cannabinoid receptor agonists. It is possible, therefore, that the rewarding properties of cannabinoids in the CPP procedure may be masked by aversive or dysphoric effects, under particular circumstances. Thus, we highlight the difficulty of drawing general conclusions on whether Δ^9^-THC and other cannabinoids have reinforcing properties in the CPP paradigm.

## Cannabinoid Effects on Self-Administration Studies

Human subjects and laboratory animals will self-administer addictive drugs by a variety of routes, including oral, intragastric, intraperitoneal, and intracranial routes. Intravenous drug self-administration has been one of the most direct approaches to study the rewarding properties of drugs of abuse in experimental animals, such as rodents or primates. In this behavioral paradigm, based on operant conditioning, animals learn to make an operant response, such as pressing a lever in an operant chamber or inserting their nose into a hole, to self-administer a reinforcer (e.g., a drug of abuse) after the completion of the reinforcement schedule requirement. A reinforcer is an event that follows a response and increases the probability of a response to reoccur (143–147). Reinforcing effects of a drug assessed by intravenous self-administration procedures in experimental animals are considered as one of the most reliable predictors of abuse potential in human subjects. The main schedules of reinforcement used in the self-administration procedure to resemble the human condition are the fixed-ratio, the progressive-ratio, and the discrete-trials schedules of reinforcement.

Briefly, under the fixed-ratio schedule, the reinforcer is delivered every time a predetermined number of responses is completed, and the delivery of a reinforcer is usually followed by a timeout period in self-administration studies to prevent the subjects from overdosing (e.g., FR1 or continuous reinforcement schedule, FR2, FR4, FR5, etc.). Data obtained from a fixed-ratio schedule provide a measure of drug intake and reinforcement efficacy. Further, under the progressive-ratio schedule, the response requirements are progressively increased after the delivery of each reinforcer, according to a predetermined progression. For example, the number of responses required to earn a nicotine infusion or food pellet on the progressive-ratio can be determined by the exponential progression [5e^(0.25 × (infusion number + 3))^ − 5] with the first two values replaced by 5 and 10, so that the response requirements for successive reinforcers are 5, 10, 17, 24, 32, 42, 56, 73, 95, 124, 161, 208, etc. Breakpoints in this schedule are typically defined as the highest response rate achieved to obtain a single reinforcer before an animal fails to complete the next ratio requirement within a predetermined time period (e.g., 60 min). Data obtained from a progressive-ratio schedule provide a measure of the motivation (i.e., incentive value) to obtain a reinforcer. Finally, in the discrete-trial schedule of reinforcement procedure, only a single injection of the drug is delivered during individual trials. The intertrial interval (ITI) can be adjusted to manipulate the influence of one injection on subsequent trials. When short ITIs are used, animals continuously self-administer a drug for long periods of time (hours or even days). When long ITIs are used, a regular circadian pattern of self-administration occurs (i.e., periods of abstinence during the light phase of the cycle alternate with periods of self-administration during the dark phase). Data obtained from a discrete-trials schedule provide a measure of the motivation to initiate drug-taking behavior. Thus, all three schedules can reliably predict abuse potential in human subjects (147).

Most drugs abused by humans, including psychostimulants, opiates, ethanol, and nicotine, support reliable and persistent self-administration behavior in drug-naïve experimental animals (148). However, for many years, it has been rather difficult to show self-administration of cannabis, Δ^9^-THC or other cannabinoid compounds in experimental animals (149–155), with the first studies showing either no effect of Δ^9^-THC (156, 157), self-administration of Δ^9^-THC only in food- or water-deprived animals (117, 158–160), or in animals that were previously pre-exposed to or trained to self-administer other drugs of abuse, such as phencyclidine, cocaine, amphetamine, ethanol, or pentobarbital (150, 155, 161–163), with not a robust effect (i.e., relatively low rates of responding). Interestingly, in the past few years, different research groups have successfully varied the parameters of self-administration procedure in order to demonstrate reliable and persistent self-administration of Δ^9^-THC or other synthetic cannabinoids in rodents or primates (see Table 3).

**Table 3:** **Cannabinoid effects on self-administration in experimental animals**.

Cannabinoid drug	Dose	Effect	Species	Reference
WIN 55,212-2	0.05–0.1 mg/kg	↑SA	CD1 mice	(164)
	0.5 mg/kg	↓SA		

Δ^9^-THC	2 and 4 mg/kg/injection	↑SA	Squirrel monkeys	(162)

WIN 55,212-2	6.25–50 μg/kg/injection	↑SA	Long-Evans rats	(165)

CP 55,940	0.1–1.6 mg/2 μl/infusion	SA	Wistar rats	(113, 114)

Δ^9^-THC	2, 4, 8 mg/kg/injection	↑SA	Squirrel monkeys	(166)

Δ^9^-THC	2–8 μg/kg/injection	↑SA	Squirrel monkeys	(167)

WIN 55,212-2	0.6, 1.2 and 1.8 mg/kg	↓SA	Wistar rats	(168)

Anandamide (AEA)	40 mg/kg/injection	↑SA	Squirrel monkeys	(169)

Methanandamide	10, 20, 40 μg/kg/injection	↑SA		(169)

Δ^9^-THC	100 ml injection of 66 or 200 pmol	SA	Sprague-Dawley rats	(170)

WIN 55,212-2	6.25 and 12.5 μg/kg/infusion	SA	CD1 mice	(171)

WIN 55,212-2	12.5 μg/kg/infusion	SA	Sprague-Dawley rats	(172)

WIN 55,212-2	12.5 μg/kg/infusion	↑SA	Lister Hooded and Long Evans rats	(173)

WIN 55,212-2	12.5 μg/kg/infusion	SA	Lister Hooded rats	(174)

2-Arachidonoylglycerol (2-AG)	0.1–100 μg/kg	↑SA	Squirrel monkeys	(175)

Δ^9^-THC	0.0032–0.032 mg/kg/infusion	SA	Rhesus monkeys	(176)

Δ^9^-THC	4 μg/kg/injection	↑SA	Squirrel monkeys	(177)

WIN 55,212-2	12.5 μg/kg/infusion	↑SA	Sprague-Dawley rats	(177)

WIN 55,212-2	12.5 μg/kg/infusion	↑SA	C57BL/6J mice	(178)

WIN55,212-2	0.01 mg/kg/infusion	↑SA	Long-Evans rats	(179)

Δ^9^-THC	0.003–0.1 mg/kg/infusion	SA	Long-Evans rats	(179)

Δ^9^-THC	4 μg/kg/injection	↑SA	Squirrel monkeys	(180)

*↑ SA: increase, ↓ SA: decrease in self-administration (SA), SA: self-administration in not higher than vehicle/saline levels*.

The first self-administration of cannabis, with a low success rate, was reported by Deneau and Kaymakcalan (156) and Kaymakcalan (157), who demonstrated acquisition of Δ^9^-THC self-administration behavior in two monkeys out of six studied, but only after withdrawal from forced automatic i.v. injections of Δ^9^-THC, when signs of physical dependence on Δ^9^-THC occurred. Naïve monkeys did not self-administer Δ^9^-THC, while one monkey exhibited Δ^9^-THC self-administration behavior following cocaine self-administration. Furthermore, in a study by Pickens and colleagues (161) where animals had been pre-exposed to phencyclidine before Δ^9^-THC self-administration, rates of responding were relative low and there was no clear evidence that responding for Δ^9^-THC could persist above vehicle control levels over repeated daily sessions. The functional state as well as the motivational state in naïve animals compared with animals that self-administer other drugs of abuse could be different, and therefore, their corresponding response could also vary accordingly (154, 181). Similarly to the above study, food deprivation was also used to initiate and subsequently maintain Δ^9^-THC self-administration. Takahashi and Singer (158, 159) reported Δ^9^-THC self-administration above placebo levels in diet-restricted rats maintained at 80% of normal body weight, under conditions where a food pellet was automatically delivered every minute. Interestingly, self-administration immediately decreased to placebo levels when food restriction was discontinued. This manipulation may also alter the motivational state of the animal, which *per se* is an inherent limitation, as it has been repeatedly shown that food restriction (or deprivation) can facilitate the initiation and maintenance of drug self-administration (160, 182–185).

Interestingly, initiation and maintenance of high rates of intravenous self-administration of low doses of Δ^9^-THC in drug-naïve squirrel monkeys was only accomplished in the past few years (166, 180, 186). In the first of these studies, low doses of Δ^9^-THC initiated and sustained high rates of intravenous self-administration in drug-naïve squirrel monkeys. Three drug naïve squirrel monkeys were used and low doses of Δ^9^-THC (1–8 μg/kg/injection) that, according to the authors, were several times lower than doses generally used in previous attempts to demonstrate Δ^9^-THC self-administration in monkeys and comparable to those delivered by an average marijuana cigarette. Furthermore, Δ^9^-THC was dissolved in a Tween-80 vehicle resulting in a clear solution that was rapidly delivered (0.2 ml injection delivered in 200 ms) in the drug-naïve animals. The self-administration behavior was rapidly extinguished either by substituting vehicle injections for Δ^9^-THC injections or by administering the CB_1_ receptor antagonist SR141716A before the session, demonstrating that this effect was mediated by direct stimulation of the CB_1_ receptors. Most recently, in a study by a different research group (176), rhesus monkeys could self-administer Δ^9^-THC alone (0.0032–0.032 mg/kg/infusion), although Δ^9^-THC alone did not maintain responding above that obtained with saline.

Importantly, Braida and colleagues also showed intracerebroventricular self-administration of Δ^9^-THC in rats under water-deprived conditions (117), while in a latter study Zangen and colleagues (170) identified the posterior ventral tegmental area and the shell of the nucleus accumbens, but not the anterior ventral tegmental area, the region dorsal to this, or the core of the nucleus accumbens, as possible brain sites for the rewarding effects of the reported intracerebral self-administration of Δ^9^-THC (170).

Synthetic cannabinoid analogs have also been used in the self-administration procedure. The most commonly used synthetic cannabinoid analog is the potent non-selective CB_1_/CB_2_ receptor agonist WIN55,212-2. Fattore and colleagues (165) showed that rats could self-administer intravenously several doses of WIN55,212-2 under food restriction. This effect was blocked by the CB_1_ receptor antagonist SR141716A, indicating that the self-administration of WIN55,212-2 was mediated by activation of the CB_1_ receptors. This finding was replicated in more recent studies by the same group using the same experimental design (173, 174). Further, Lecca and colleagues (172) reported self-administration of WIN55,212-2 in rats following a different experimental protocol from that of the above mentioned studies. In their study, rats were not food-restricted, but they were maintained on a daily ratio of 20 g of food, made available at the end of each self-administration session.

In a most recent study (179), male Long-Evans rats were trained to self-administer WIN55,212-2 (0.01 mg/kg/infusion) on a fixed ratio 3 schedule. Dose–effect curves for WIN55,212-2 were determined, followed by vehicle substitution and a dose–effect curve with Δ^9^-THC. WIN55,212-2 self-administration was acquired; however, substitution with Δ^9^-THC did not maintain responding above vehicle levels. WIN55,212-2’s reinforcing effects were CB_1_ receptor-mediated, as they were dose-dependently attenuated by SR141716A. As authors indicated, the lack of substitution with Δ^9^-THC seen in this study is problematic and may suggest that WIN55,212-2 self-administration may be of limited usefulness as a screening tool for detection of the reinforcing effects of potential cannabinoid medications (179).

Importantly, Martellotta and colleagues showed intravenous self-administration of WIN55,212-2 in mice in a dose-dependent manner (164). This effect was also blocked by pre-treatment with the CB_1_ receptor antagonist SR141716A, indicating the direct involvement of the CB_1_ receptors. Self-administration of WIN55,212-2 in mice under a fixed- and a progressive-ratio schedule of reinforcement was also shown recently (178), an effect that was blocked by systemic administration of the hypocretin receptor-1 (Hcrtr-1) antagonist SB334867. This role of Hcrtr-1 in the reinforcing and motivational properties of WIN55,212-2 was confirmed in Hcrtr-1 knockout mice (178).

The same experimental protocol as Martellota and colleagues (164) was also used by another research group (187) to study the reinforcing effects of WIN55,212-2 in CB_1_ knockout mice. The genetically modified mice did not self-administer WIN55,212-2. In another study, drug-naïve mice self-administered the synthetic CB_1_ receptor agonist WIN55,212-2 and the Δ^9^-THC derivative HU-210 (188). However, it should be emphasized that these studies have an important inherent limitation as 1-day experimental tests were used and the animals were severely restrained. Thus, validity of these data is questionable and difficult to correlate with drug addiction in humans, which is a chronic state or even compare with chronic self-administration procedures in animals under baseline conditions (i.e., no restraint). Furthermore, since the animals were severely restrained, the reported self-administration may be affected by analgesic or anxiolytic effects resulting in a reduction of pain or stress produced by the restrain.

Interestingly, both AEA (as well as its metabolically stable synthetic analog methanandamide) (169) and 2-AG (175) are intravenously self-administered by squirrel monkeys, although four out of six squirrel monkeys used in the first study (169) had a history of Δ^9^-THC or methohexital self-administration. Similarly, in the more recent study indicating 2-AG self-administration, the researchers used monkeys with either a history of AEA self-administration or a history of nicotine self-administration (175). Interestingly, however, the reinforcing effects of AEA and 2-AG appear to be mediated by cannabinoid CB_1_ receptors, since daily pre-treatment with SR141716A resulted in complete blockade of AEA or 2-AG self-administration behavior. It is also noteworthy that in both studies, the authors report rates of responding comparable with those maintained under the same conditions by cocaine or Δ^9^-THC. More importantly, there is also evidence that treatment with the FAAH inhibitor URB597 shifts the AEA self-administration dose–response curve to the left, indicating that AEA has rewarding effects even in lower doses (189).

Further, only a few studies have focused on the intracranial self-administration of Δ^9^-THC or other cannabinoid analogs by experimental animals. Intracerebral administration of the potent non-selective CB_1_/CB_2_ receptor agonist CP-55,940 was shown in rats in a free-choice procedure (114). This effect was antagonized by the CB_1_ receptor antagonist SR141716A, indicating that it was specifically mediated by CB_1_ receptors. However, one limitation of this study is that the animals were water-deprived and water was concurrently delivered with each infusion. This may have altered the motivational state of the animals, provoking the self-administration response. In a previous study, CP-55,940 was not self-administered by rhesus monkeys (155).

In summary, most attempts to obtain a robust self-administration of Δ^9^-THC or other synthetic cannabinoids, under regular experimental conditions (i.e., drug-naïve unrestrained animals, and not food deprived), have been unsuccessful or partly successful. Only a limited number of studies report a robust procedure for cannabinoid self-administration either in a limited number of squirrel monkeys or intracerebrally in rodents. This is in accordance with other behavioral studies on rewarding and reinforcing effects of cannabinoids (i.e., ICSS, CPP) and illustrates the differential status of cannabinoids as atypical drugs of abuse.

## Cannabinoid Effects on Reinstatement Procedures

A procedure used to study cue-, context-, drug-, or stress-induced reinstatement of drug seeking is hypothesized to be a putative model of relapse to drug seeking in humans. Animals learn to self-administer a drug for a period of time, in the same manner as during the self-administration procedure. Drug-reinforced lever responding is then extinguished, and reinstatement of drug-seeking behavior is subsequently triggered by a priming injection of a compound (drug-induced), a cue (or context) previously associated with the self-administration of the drug (cue- or context-induced), or a stressor (stress-induced reinstatement) (147). The reinstatement model of relapse to drug-seeking behavior is uniquely responsive to drugs with addictive properties. Only drugs which support drug-seeking and drug-taking behaviors can initiate or trigger relapse in the reinstatement model. Especially, compelling is the fact that cross-priming (from one class of addictive drug to another) is seen in this model (190).

Little work has been done with cannabinoids *per se* in this model. However, existing literature in cannabinoid research indicates that most of the reinstatement studies conducted with cannabinoid compounds test for the cross-priming effect (i.e., the interactions between cannabinoid compounds and other drugs of abuse in inducing reinstatement of drug seeking) and is suggestive of cannabinoids fitting the same pattern as other addictive drugs in these procedures [for reviews, see Ref. (191–196)]. In many cases, cannabinoids trigger reinstatement of drug-seeking behavior in animals behaviorally extinguished from intravenous drug self-administration behavior and, thus, pharmacologically detoxified from their self-administered drug. Thus, in most cases, either different drug of abuse has been used before extinction (e.g., cocaine, heroin, morphine) or the drug-induced reinstatement is triggered by cannabinoids or *vice versa* (186, 197) (please see Table 4).

**Table 4:** **Cannabinoid effects on reinstatement of drug-seeking behavior in experimental animals**.

Priming drug/cue/stress factor	Dose	Self-administration drug	Dose	Effect	Species	Reference
**DRUG-INDUCED REINSTATEMENT**						
HU210	20 and 100 μg/kg	Cocaine	0.5 mg/kg	√	Male Wistar rats	(198)

HU210	20 μg/kg	Heroin	50 μg/kg/infusion	√	Male Wistar rats	(199)
Heroin	0.25 mg/kg			√		
SR1412716A + Heroin	3 mg/kg + 0.25 mg/kg			–		

WIN 55,212-2	0.15 and 0.3 mg/kg	Heroin	0.03 mg/kg/injection	√	Male Lister Hooded rats	(200)
CP 55,940	0.05 and 0.1 mg/kg			√		
Δ^9^-THC	0.1–1.0 mg/kg			No effect		
SR1412716A + Heroin	0.3 mg/kg + 0.1 mg/kg					

Methamphetamine	1 mg/kg	Methamphetamine	0.02 mg/kg/infusion	√	Male Wistar rats	(201)
Δ^8^-THC	0.32–3.2 mg/kg			No effect		
SR1412716A + Δ^8^-THC	3.2 mg/kg + 1 mg/kg			Decreased lever press responses		

WIN 55,212-2	0.25 and 0.5 mg/kg^−1^	WIN 55,212-2	12.5 μg/kg/inf^−1^	√	Male Long Evans rats	(202)
Heroin	0.5 mg/kg^−1^			√		
Cocaine	10 mg/kg^−1^			–		
SR 141716A	mg/kg^−1^			No effect		
SR + WIN/heroin	1 mg/kg^−1^			Blocks WIN/heroin-reinstatement		
Naloxone + WIN/heroin				Blocks WIN/heroin-reinstatement		

Δ^9^-THC	1 mg/kg	Beer	4.5% ethanol v/v	√	Male Wistar rats	(203)
		Near-beer	<0.5% ethanol v/v	√		

Heroin	0.1 mg/kg	Heroin	0.03 mg/kg/inf	√	Male Lister	(204)

WIN 55,212-2	0.15 and 0.3 mg/kg			√	Hooded rats	
CP55,940	0.05 and 0.1 mg/kg			√		
SR141716A	0.3 mg/kg			No effect		
SR + WIN				–		
SR + CP				–		
SR + Heroin				–		

Methamphetamine	0.01–1.78 mg/kg	Methamphetamine	0.1 mg/kg/infusion	√	Male Spreague- Dawley rats	(205)
AM251 + Methamphetamine	0.032–0.32 mg/kg + 0.01–1.78 mg/kg			No effect on methamphetamine		

Methamphetamine	mg/kg	Methamphetamine	0.02 mg/0.1 ml/infusion	√	Male Wistar rats	(206)
HU210	10–32 μg/kg per side			√		
AM251 + HU210	32 μg/kg per side + 10–32 μg/kg per side			–		

URB597	0.3 mg/kg	Δ^9^-THC	1 and 4 μg/kg	No effect	Male Squirrel monkeys	(189)
		Anandamide	3 and 56 μg/kg	No effect		
		cocaine	1 and 30 μg/kg	No effect		

Δ^9^-THC	40 μg/kg (end of session)	Δ^9^-THC	10, 20, 40 and 80 μg/kg (total end of session doses)	√ (except for the lowest dose)	Male Squirrel monkeys	(186)
SR141716A + Δ^9^-THC	0.3 mg/kg (start) + 40 μg/kg (end of session)			–		
Natlrexone + Δ^9^-THC	0.1 mg/kg (start) + 40 μg/kg (end of session)			–		

Nicotine	0.03 mg/kg/inf	Nicotine	0.03 mg/kg/inf	√	Male Lister Hooded rats	(207)
AM251 + Nicotine	1, 3, 10 mg/kg + 0.2 mg/kg			–		

WIN55,212-2	0.15 or 0.3 mg/kg^−1^	WIN55,212-2	12.5 mg kg^−1^ per infusion	Intact female rats exhibited stronger reinstatement than males and ovariectomized females	Female Lister Hooded rats	(208)
					Female ovariectomized Lister Hooded rats	
					Male Lister Hooded rats	

WIN55,212-2	0.15–0.3 mg kg(−1)	Heroin	30 μg kg^−1^/infusion	√	Male Lister Hooded rats	(209)
Naloxone	0.1–1 mg kg(−1)			–		
SR141716A	0.3–3 mg kg(−1)			–		

Selective adenosine A(2A) receptor antagonist MSX-3	1 and 3 mg/kg	Δ^9^-THC	4 μg/kg	No effect	Male Squirrel monkeys	(210)
**CUE-INDUCED REINSTATEMENT**						
House light and click/light signal	3 mg/kg	Heroin	50 μg/kg/infusion	√	Male Wistar rats	(199)
SR1412716A				–		

WIN 55,212-2	0.3, 1, and 3 mg/kg (daily during 5-day extinction)	Cocaine	0.25 mg/kg/inf	√ (0.3 mg/kg)	Male Wistar rats	(211)

Δ^9^-THC	5 mg/kg/day, perinatal	Ethanol	3% v/v	No effect	Primiparous Wistar female rats	(212)
Δ^9^-THC + ethanol	5 mg/kg/day + 3% v/v			No effect		
SR-141716A	0.3–3.0 mg/kg			–		

AM404	0.4, 2 and 10 mg/kg	Ethanol	10% v/v	No effect	Male Wistar rats	(213)

AM251	32 μg/kg per side	Methamphetamine	0.02 mg/0.1 ml/infusion	–	Male Wistar rats	(206)

Cue with or without end of session Δ^9^-THC	40 μg/kg (end of session)	Δ^9^-THC	10, 20, 40 and 80 μg/kg (total end of session doses)	√	Male Squirrel monkeys	(186)
SR141716A + Cue	0.3 mg/kg (start)			–		

Nicotine	0.03 mg/kg/inf	Nicotine	0.03 mg/kg/inf	√	Male Lister Hooded rats	(207)
AM251 + Nicotine	1, 3, 10 mg/kg + 0.2 mg/kg			–		

Tone or Light Cue		WIN55,212-2	12.5 mg kg^−1^ per infusion	Intact female rats exhibited stronger reinstatement than males and ovariectomized females	Female Lister Hooded rats	(208)
					Female ovariectomized Lister Hooded rats	
					Male Lister Hooded rats	
**STRESS-INDUCED REINSTATEMENT**						
*(foot-shock)*		Ethanol	3% v/v		Primiparous Wistar female rats	(212)
Δ^9^-THC	5 mg/kg/day, perinatal			No effect		
Δ^9^-THC + ethanol	5 mg/kg/day + 3% v/v			No effect		
SR-141716A	0.3–3.0 mg/kg			No effect		

*√ Induced reinstatement; – blocked reinstatement/blocked effects of CB_1_ agonists*.

CB_1_ receptors have been found to play a critical role in mediating reinstatement of previously extinguished drug-seeking behavior upon re-exposure to the drug or drug-associated cues. The neuroanatomical bases as well as the neuronal mechanisms of the relapse-promoting effects of CB_1_ receptor agonists or the relapse-attenuating effects of CB_1_ receptor antagonists are still poorly understood, although interactions of the endogenous cannabinoid system with afferent glutamatergic and possibly dopaminergic projections to the nucleus accumbens are most likely involved (214).

Systemic injections of the potent CB_1_ receptor agonist HU-210 dose-dependently reinstate cocaine-seeking behavior in laboratory rats behaviorally extinguished from intravenous cocaine self-administration (198). Systemic injections of HU-210 also reinstate heroin-seeking behavior in laboratory rats behaviorally extinguished from intravenous heroin self-administration (199). Interestingly, however, the same research group found that the CB_1_ cannabinoid receptor antagonist SR-141716A blocked reinstatement to drug-seeking behavior triggered by cocaine, heroin, or cocaine-associated environmental cues, but not reinstatement induced by exposure to stress, suggesting a potential role for cannabinoid antagonists in the treatment of addiction. Cue-induced reinstatement to cocaine seeking has also been found when rats were administered with different doses of WIN55,212-2 (0.3, 1, and 3 mg/kg) during a 5-day extinction period. In this case, the lowest dose of WIN55,212-2 (0.3 mg/kg) induced the highest resistance to extinction and reinstatement (i.e., the highest responding at the active lever during conditioned-reinstatement) (211).

Interestingly, however, squirrel monkeys did not self-administer the FAAH inhibitor URB597, and the drug did not promote reinstatement of extinguished drug-seeking behavior previously maintained by Δ^9^-THC, anandamide, or cocaine (189). Further, reinstatement to Δ^9^-THC-seeking behavior does not seem to be affected by striatal adenosine receptors, as the selective adenosine A(2A) receptor antagonist MSX-3 (1 mg/kg) neither promoted reinstatement of extinguished drug-seeking behavior nor altered reinstatement of drug-seeking behavior by non-contingent priming injections of Δ^9^-THC (210).

In another study using psychostimulants (201), following 12 days of self-administration of methamphetamine (METH), under extinction conditions, METH-priming or re-exposure to cues previously paired with METH infusion triggered reinstatement of METH seeking. The cannabinoid CB_1_ receptor antagonist SR141716A blocked this effect, while administration of the cannabinoid agonist, Δ^8^-tetrahydrocannabinol (Δ^8^-THC), had no effect by itself, and co-administration of the Δ^8^-THC and METH at small doses reinstated the drug-seeking behavior. Interestingly, Δ^8^-THC attenuated the effects of the reinstatement-inducing dose of METH, but enhanced the effect of cues. Either given repeatedly during the extinction or singly, 24 h before the first METH-priming or cues challenge, Δ^8^-THC suppressed the reinstatement (201). These results suggest that the endocannabinoid system may be involved in the reinstating effects of METH-priming and cues. A follow-up study by the same group examined whether the reinstatement involves interactions between CB_1_ and nicotinic acetylcholine receptors (nAChRs) in the reinstatement of METH-seeking behavior (206). Systemic and intracranial administration of the potent CB_1_ receptor agonist HU210 into the nucleus accumbens core and prelimbic cortex reinstated METH-seeking behavior. The reinstatement caused by the systemic HU210 treatment was attenuated by intracranial administration of the CB_1_ receptor antagonist AM251 into the regions mentioned above, while reinstatement induced by the METH-associated cues and METH-priming injection was also attenuated by intracranial administration of AM251 in each region. Interestingly, in these regions, the attenuating effects of AM251 on the reinstatement induced by each stimulus were blocked by the intracranial administration of mecamylamine, a non-selective nAChR antagonist, but not by scopolamine, a muscarinic ACh receptor (mAChR) antagonist. Moreover, the intracranial administration of DHβE, an α4β2 nAChR antagonist, but not MLA, an α7 nAChR antagonist, into each region blocked the AM251-induced attenuation of the reinstatement. These findings suggest that reinstatement (or relapse in humans) to MAP-seeking behavior may be due to two steps: inhibition of ACh transmission by the activation of cannabinoid CB_1_ receptors and inactivation of α4β2 nAChRs (206). On the contrary, another study using AM251 did not modify METH-induced reinstatement of METH-seeking behavior (205).

The effects of the selective CB_1_ receptor antagonist AM251 have also been tested in nicotine-seeking behavior (207), where it has been found to dose-dependently (1–10 mg/kg) attenuate the reinstatement effects produced by both a nicotine priming dose (0.2 mg/kg) and its contingently presented cues.

Similarly to the studies presented above, Fattore and colleagues (200) showed that intraperitoneal priming injections of the potent non-selective CB_1_/CB_2_ receptor agonists WIN 55,212-2 (0.15 and 0.3 mg/kg) and CP 55,940 (0.05 and 0.1 mg/kg), but not Δ^9^-THC (0.1–1.0 mg/kg), effectively restored heroin-seeking behavior. In the same study, intraperitoneal priming injection of the CB_1_ receptor antagonist SR141716A (0.3 mg/kg) did not reinstate responding, but completely prevented heroin-induced reinstatement of drug-seeking behavior. Moreover, heroin-seeking behavior was still present for a few days following cannabinoid primings, indicating a long-lasting effect of cannabinoids on responding for heroin. These findings indicate that relapse to heroin after an extended drug-free period is triggered by cannabinoid agonists and that SR 141716A prevents drug-seeking behavior, suggesting that the use of the cannabinoid antagonists could have some therapeutic benefits in heroin-induced relapse (200). A follow-up study also presented similar findings (204). In continuation of the above study, a very interesting study from the same group showed that rats previously trained to intravenously self-administer the CB_1_ receptor agonist WIN 55,212-2 (12.5 μg/kg/inf) showed reinstatement in WIN 55,212-2-seeking behavior after priming injections of either the previously self-administered CB_1_ agonist (0.25 and 0.5 mg/kg) or heroin (0.5 mg/kg), but not cocaine (10 mg/kg), following 3 weeks of extinction. The selective CB_1_ receptor antagonist SR 141716A (0.3 mg/kg) did not reinstate responding when given alone, but completely prevented the cannabinoid-seeking behavior triggered by WIN 55,212-2 or heroin primings. Further, the non-selective opioid antagonist naloxone (1 mg/kg) had no effect on operant behavior *per se*, but significantly blocked cannabinoid- and heroin-induced reinstatement of cannabinoid-seeking behavior (202).

Most recently, WIN55,212-2-induced reinstatement of heroin-seeking behavior was significantly attenuated by naloxone (1 mg/kg) and rimonabant (3 mg/kg) and fully blocked by co-administration of sub-threshold doses of the two CB_1_ receptor antagonists. Moreover, contrary to immediate (1 day) or delayed (90 days) drug substitution, rats readily self-administered WIN when access was given after 7, 14, or 21 days of extinction from heroin, and showed a response rate that was positively correlated with the extinction period (209). Taken together, this set of data suggests some strong interactions between the cannabinoid and opioid systems in relapse mechanisms.

In relation to ethanol/alcohol-seeking behavior, Δ^9^-THC (1 mg/kg) significantly reinstated responding, previously reinforced with beer or near-beer (low alcohol beer) (203), while the anandamide transport inhibitor AM404 did not affect cue-induced reinstatement of alcohol-seeking behavior (213). On the other hand, perinatal administration of Δ^9^-THC (5 mg/kg, daily) either alone or in combination with ethanol (3% v/v) did not affect alcohol self-administration or alcohol seeking in any of the rat groups, while SR141716A (0.3–3.0 mg/kg) significantly reduced lever pressing for ethanol and blocked conditioned reinstatement of alcohol seeking, although the same doses of SR141716A failed to block foot-shock stress-induced reinstatement of alcohol seeking (212).

Finally, sex differences and ovarian hormones also appear to play a role in modulating cannabinoid-seeking behavior after exposure to drug priming or drug-asociated cues. In the study by Fattore and colleagues (208), after a priming dose of 0.15 or 0.3 mg/kg WIN55,212-2, intact female rats exhibited stronger reinstatement than males and ovariectomized females. Responses of intact female rats were higher than those of male and ovariectomized rats even after priming with a drug-associated visual or auditory cue, or a WIN55,212-2 + Cue combination (208).

In summary, the majority of the studies presented show that CB_1_ receptor agonists or endocannabinoid enhancers tend to promote either drug-, or cue-, or stress-induced reinstatement of drug-seeking behavior either to cannabinoid compounds or to other drugs of abuse. Overall, the above findings indicate that the endocannabinoid system, and in particular the CB_1_ receptors, play an important role in the processes underlying reinstatement to different drugs of abuse, such as psychostimulants (e.g., cocaine, methamphetamine, and nicotine), opioids (e.g., heroin) and alcohol. Further research will help clarify the mechanisms underlying these drug interactions and cross-priming effects in reinstatement processes.

## Conclusion

Although the euphorigenic properties of cannabis preparations have been appreciated by humans for centuries, only the last years we have acquired the experimental tools to evaluate cannabinoid reward and abuse liability in experimental animals. It is now clear that cannabinoids exert emotional and motivational effects in experimental animals and can activate the same reward circuits in the brain and produce drug reinforcement/drug-seeking behavior, although under more limited conditions. The rewarding properties of Δ^9^-THC are clearly shown by a decrease in brain-stimulation reward thresholds and self-administration behavior. However, CB_1_ receptor agonists and endocannabinoid modulators (indirect agonists) do not affect the reinforcing efficacy of brain stimulation and are self-administered basically under particular experimental conditions. Moreover, contrasting findings have been shown in the CPP paradigm, where cannabinoids produce both positive (rewarding) and negative (aversive) effects, depending on the specific experimental procedures followed. Beyond any doubt, cannabinoids and the endocannabinoid system appear to be involved in reinstatement of extinguished self-administration of several drugs of abuse. Much remains to be done before we fully understand the actions of cannabinoids in critical areas of the reward circuit that mediate both rewarding and aversive phenomena and relapse mechanisms. Furthermore, since new cannabinoid-related medications are being developed, there will be a need to assess their potential rewarding actions and abuse liability, using the animal models and experimental procedures described here. The fact, for example, that enhancement of endocannabinoid neurotransmission does not increase brain reward, neither produces reward-related behaviors makes the drugs that directly affect endocannabinoid levels promising therapeutics, with less unwanted side-effects and minimal abuse potential.

## Conflict of Interest Statement

The authors declare that the research was conducted in the absence of any commercial or financial relationships that could be construed as a potential conflict of interest.
